# The First Sertoli Cell Tumor of the Adrenal Gland is Potentially Associated with Arterial Hypertension

**DOI:** 10.5812/ijem-156823

**Published:** 2024-10-30

**Authors:** Sara Ivanis, Milan Marinkovic, Milan Jovanovic, Matija Buzejic, Marija Milinkovic, Zlatibor Loncar, Vladan Zivaljevic, Branislav Rovcanin

**Affiliations:** 1 Clinic for Endocrine Surgery, University Clinical Centre of Serbia, Belgrade, Serbia; 2 Faculty of Medicine, University of Belgrade, Belgrade, Serbia; 3 Department of Pathology, University Clinical Centre of Serbia, Belgrade, Serbia; 4 Emergency Center, University Clinical Centre of Serbia, Belgrade, Serbia

**Keywords:** Sertoli Cell Tumor, Adrenal Gland, Laparoscopic Surgery, Case Report

## Abstract

**Introduction:**

Sertoli cell tumors are rare sex cord-stromal tumors, accounting for less than 1% of primary testicular tumors. They typically arise in the testes and ovaries, with other localizations being uncommon. We present the case of a Sertoli cell tumor in the adrenal gland, which, to our knowledge, is the first reported in the literature.

**Case Presentation:**

A 44-year-old male patient was admitted to the clinic for endocrine surgery for laparoscopic surgery of a right adrenal gland incidentaloma measuring 57 × 47 × 59 mm, discovered during a routine abdominal ultrasonography. The patient had a history of hypertension but no other comorbidities. Biochemical and physical examinations revealed no signs of hypercortisolism. Urinary metanephrine and normetanephrine levels were within normal limits. A right laparoscopic adrenalectomy was performed, and a 5 cm tumor was identified without evidence of locoregional invasion. Pathological examination confirmed a Sertoli cell tumor of the adrenal gland. Immunohistochemical analysis revealed positive staining for vimentin, steroidogenic factor 1 (SF1), and beta-catenin, while chromogranin A, hCG, PSA, and TTF1 were negative. The Ki-67 index was 3%. The patient was subsequently referred to a urologist, where testicular ultrasonography showed no abnormalities. There were no signs of recurrence during a 15-month follow-up period. Additionally, the patient’s biannual antihypertensive treatment was discontinued by a cardiologist 1.5 months post-surgery.

**Conclusions:**

Sertoli cell tumors are an exceptionally rare entity. To our knowledge, this is the first reported case of a primary Sertoli cell tumor originating in the adrenal gland. Given their potential for malignancy, regular follow-up and additional diagnostic evaluations may be necessary. Laparoscopic adrenalectomy appears to be a suitable definitive treatment for this condition.

## 1. Introduction

Sertoli cell tumors are a type of sex cord-stromal tumor, accounting for less than 1% of primary testicular tumors (1). They are classified into Sertoli cell tumor, not otherwise specified (NOS), the sclerosing variant, and the large cell calcifying variant (2). The average age at diagnosis is approximately 45 years, although the large cell calcifying variant primarily occurs in patients younger than 20 years (3). While these tumors are mostly sporadic, they have been associated with Peutz–Jeghers syndrome and Carney complex, in which cases they often present bilaterally (4). Approximately 10% of Sertoli cell tumors are considered malignant (5). They are typically asymptomatic but may present with pain or gynecomastia (6). Beyond the ovarian variant, extratesticular localization of Sertoli cell tumors is extremely rare (7).

We present the case of a Sertoli cell tumor in the adrenal gland, which, to our knowledge, is the first described in the literature.

## 2. Case Presentation

A 44-year-old male Caucasian patient with a BMI of 29.97 kg/m², non-smoker, was admitted to the Clinic for Endocrine Surgery following the discovery of a right adrenal gland incidentaloma during a routine echotomographic examination of the abdomen. He denied symptoms such as abdominal pain, headache, or palpitations and did not report any comorbidities aside from a history of arterial hypertension, with the highest recorded blood pressure values reaching 160/100 mmHg. He had been treated with a combination of a calcium antagonist, an ACE inhibitor, and a diuretic for two years prior to surgery. 

Physical examination revealed no visible signs of hypercorticism. Gonadal development during puberty was typical, with no evidence of gynecomastia or precocious puberty, and he reported no fertility issues. Blood count, basic biochemistry, renal function, and thyroid function tests were all within the reference range. Biochemical evaluation showed normal cortisol (421 nmol/L), ACTH (43.7 ng/L), and urinary metanephrine levels (metanephrine 17 mcg/24h, normetanephrine 35.5 mcg/24h), with appropriate suppression observed during dexamethasone testing (DEX) (36 nmol/L) (Table 1). 

**Table 1.** Significant Laboratory Findings Before and After Surgery

Variables	Cortisol, (nmol/L)	ACTH, (ng/L)	DEX, (nmol/L)	Urin Metanephrine, (mcg/24h)	Urin Normetanephrine, (mcg/24h)	Chromogranin A, (ng/mL)
**Normal range**	138 - 744	9.6 - 49.7	< 50	< 31	< 84	< 108
**Preoperatively**	421	43.7	36	17	35.5	60
**Postoperatively**	486	47	-	-	-	-

**Figure 1.**
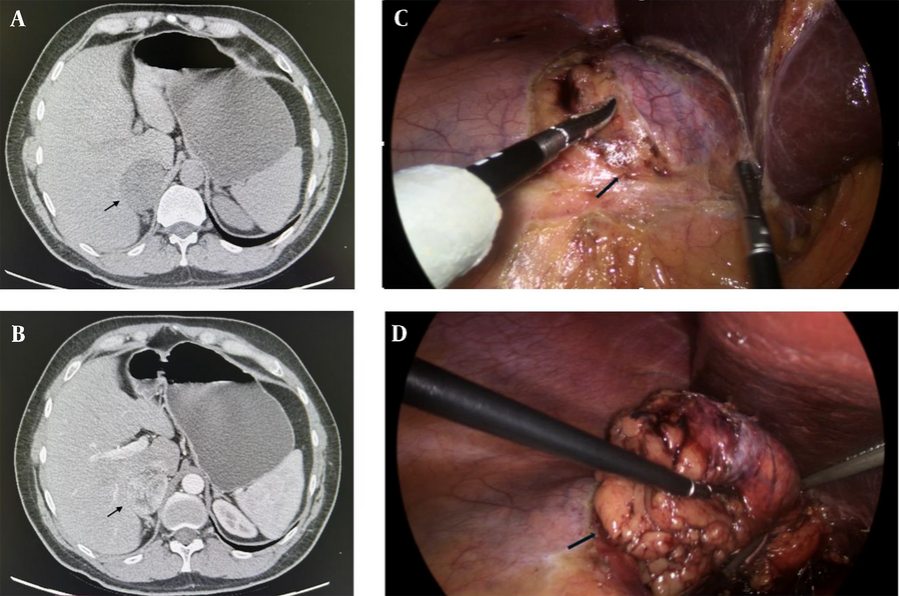
A and B, multi-slice computed tomography (MSCT) scan of the abdomen, transversal section, arrows show a right adrenal gland tumor measuring 57 × 47 × 59 mm (arrow). A, both native; and B, arterial phases are shown; C and D, intraoperative finding; C, dissection phase- exposition of the right adrenal gland tumor (arrow); D, end of dissection and extraction of the tumor a right adrenal gland tumor measuring 5 cm (arrow).

Abdominal multi-slice computed tomography (MSCT) confirmed a well-defined, regular-shaped tumor in the right adrenal gland measuring 57 × 47 × 59 mm, with a density of 2 Hounsfield Units (HU). The tumor displayed unclear post-contrast opacification with mild washout (Figure 1A and B). No focal changes were observed in the contralateral adrenal gland or retroperitoneal lymph nodes. 

The patient subsequently underwent a right laparoscopic adrenalectomy under general endotracheal anesthesia. Intraoperatively, a 5 cm mass originating from the right adrenal gland was identified. Compared to usual benign adrenal tumors, the mass exhibited a slightly firmer consistency (Figure 1C and D). The patient experienced an uneventful postoperative course and was discharged three days after surgery. 

**Figure 2.**
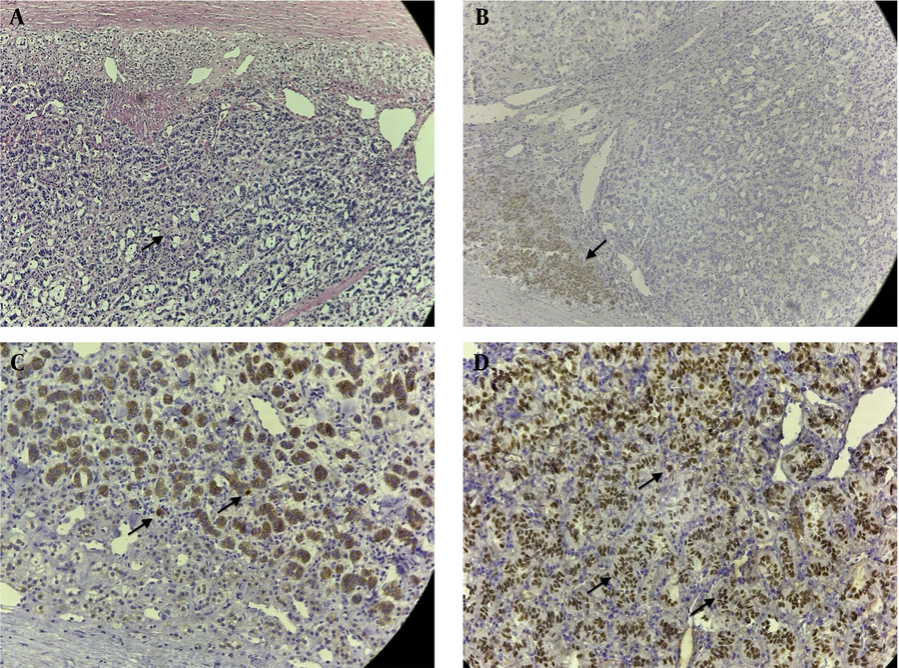
A, histology and immunostaining: Hematoxylin and eosin stain; tubular tumor tissue (arrow); and B, remains of the adrenal gland cortex, magnification, 100× which is positive for melan A, with remains of the adrenal gland cortex (arrow); C, magnification, 100× and tumor cells positive for beta catenin (arrow); D, magnification, 200× and positive for steroidogenic factor 1 (arrow), magnification 200×.

Pathological examination of the specimen revealed a Sertoli cell tumor of the adrenal gland. The immunohistochemical profile showed positivity for vimentin, steroidogenic factor 1 (SF1), beta catenin, and CD56, while chromogranin A, hCG, PSA, and TTF1 were negative, with a Ki67 proliferation index of 3%. (Figure 2A - D). 

Further urological evaluation, including testicular ultrasound, revealed no abnormalities. A postoperative abdominal MSCT scan was normal. During follow-up, the patient became normotensive, leading to the discontinuation of antihypertensive treatment by the cardiologist 1.5 months after surgery. Biochemical evaluations post-surgery demonstrated normal blood ACTH (47 nmol/L) and cortisol (486 nmol/L) levels, without the need for replacement therapy. No signs of disease recurrence were observed during a 15-month follow-up period.

## 3. Discussion

Sertoli cell tumors are rare neoplasms, constituting less than 1% of primary testicular tumors. Extratesticular occurrences are even rarer (8). While these tumors can occur in the ovaries, other primary sites have not, to our knowledge, been documented (9). Sertoli cell tumors may present with symptoms such as pain or gynecomastia, both of which were absent in our patient’s case (10). The diagnosis of Sertoli cell tumors relies on clinical manifestations, imaging techniques, and histopathological findings. Performing a biopsy for these tumors is controversial (6). Radical orchiectomy is the standard treatment for most primary tumors, but testis-sparing surgery may be an option for benign lesions. In cases of metastatic disease, a combination of surgical resection, systemic therapy, and radiation therapy may be considered (11). In our patient’s case, a right laparoscopic adrenalectomy was performed. At that stage, a Sertoli cell tumor was not suspected due to the tumor’s unusual localization. However, complete resection of the tumor was achieved, and no signs of locoregional invasion were observed during surgery.

There are no established guidelines for adjuvant therapy for Sertoli cell tumors of the testis. Aromatase inhibitors can be administered in cases of gynecomastia (12). However, adjuvant chemotherapy is recommended for advanced ovarian Sertoli cell tumors (13).

Macroscopically, Sertoli cell tumors are typically hard, well-defined, and white, yellow, or tan in color. Hemorrhagic or cystic areas may be present. This correlated with our case, which involved a 5 cm brownish tumor with cystic segments. Microscopically, Sertoli cell tumors usually exhibit a tubular pattern with minimal stroma. Immunohistochemically, they are positive for vimentin and cytokeratins (14).

*CTNNB1* gene mutations and immunohistochemical positivity for beta-catenin have been identified in approximately 60 - 70% of the NOS subtype, which is more common in benign tumors than in those with malignant characteristics (15). Steroidogenic factor-1 expression has been noted in both sex-cord stromal tumors of the testis and ovarian Sertoli cell tumors (16). These findings align with our case, which demonstrated tubular structures with focal myxoid stroma and well-differentiated cells with a low Proliferative Index (Ki67: 3%).

Immunohistochemically, the tumor was positive for vimentin, SF1, CD56, and beta-catenin, which aligns with findings reported in the literature. Markers used to exclude other tumors or potential metastases to the adrenal gland, such as chromogranin A, hCG, PSA, and TTF1, were negative. 

The most common metastatic sites for Sertoli cell tumors are retroperitoneal lymph nodes, lungs, bones, and inguinal lymph nodes. A systematic review and meta-analysis by Grogg et al. identified age older than 27.5 years, tumor diameter larger than 24 mm, presence of necrosis, extension to the spermatic cord, angiolymphatic invasion, and a high mitotic index as statistically significant variables associated with metastatic disease (17). According to current WHO guidelines, tumor size greater than 5 cm, the presence of hemorrhage, necrosis, lymphovascular invasion, and pleomorphism are considered risk factors for malignant potential. These characteristics were absent in our case, aside from the size of the tumor. However, the only definitive proof of malignancy is the occurrence of metastasis (15, 18).

While benign Sertoli cell tumors generally have a favorable prognosis, the prognosis worsens in cases of metastatic disease. Metastatic relapse has been reported after a median of 12 months, and the overall median survival of patients with metastatic disease is 20 months (17). Some articles document metastases occurring even 10 years after the initial surgery (19, 20). During the follow-up period, no signs of locoregional recurrence or dissemination of the disease were observed in our patient. He was also examined by a urologist, who found no abnormalities in the testes. 

A notable observation was the discontinuation of antihypertensive therapy shortly after surgery, suggesting a possible link between Sertoli cell tumor metabolism and the pathogenesis of arterial hypertension. However, the precise pathophysiological mechanism underlying the association between hypertension and Sertoli cell tumors remains unclear. To date, few cases in the literature have linked Sertoli cell tumors to hypertension. Two cases of renin-producing Sertoli cell tumors in female patients have been documented, both presenting with hypertension (21, 22). In our patient, the possibility of hyperaldosteronism was excluded preoperatively by the endocrinologist, making this mechanism an unlikely explanation for the tumor’s association with hypertension. Furthermore, pheochromocytoma was ruled out based on normal metanephrine, normetanephrine, and Chromogranin A levels. While biochemically silent pheochromocytomas, although rare, could account for the patient’s hypertension, pathological examination of the specimen ruled out pheochromocytoma as a diagnosis (23).

The limitations of this study stem from the lack of comparable cases of Sertoli cell tumors in the adrenal gland due to the rarity of this tumor's location. Additionally, the patient remains under follow-up due to the uncertain nature of the tumor and its potential for malignancy.

In conclusion, further research is necessary to expand knowledge about the clinical behavior of these tumors. As tumors originating from Sertoli cells may have malignant potential, regular patient follow-up and additional diagnostic tests are warranted. To our knowledge, this is the first case of a primary Sertoli cell tumor of the adrenal gland described in the literature. Based on our experience, the laparoscopic approach for adrenalectomy can be considered a method of choice for the definitive management of this type of tumor.

## Data Availability

Further data are available from the corresponding author upon reasonable request.
